# Emergency medical teams in WHO’s Western Pacific Region

**DOI:** 10.5365/wpsar.2023.14.6.1184

**Published:** 2025-07-28

**Authors:** Sean T Casey, Erin E Noste, Anthony T Cook, David Muscatello, David James Heslop

**Affiliations:** a World Health Organization Regional Office for the Western Pacific, Manila, Philippines.; b School of Population Health, Faculty of Medicine and Health, University of New South Wales, Sydney, New South Wales, Australia.; c Department of Emergency Medicine, University of California San Diego, San Diego, California, United States of America.; d National Critical Care and Trauma Response Centre, Darwin, Northern Territory, Australia.

## Abstract

This regional analysis aims to provide a comprehensive review of emergency medical team development and action in health emergency response in the Western Pacific Region from 2010 to 2024. It details national, subregional and regional efforts to strengthen health emergency preparedness, response and resilience; it notes challenges faced by the teams in these efforts; and it provides examples that could be adopted or adapted to strengthen their development and action around the world. Emergency medical teams are critical components of national, regional and global health emergency workforces, enabling rapid, high-quality and self-sufficient responses to health emergencies domestically or internationally. They comprise clinical, mental health, public health, logistics and water/sanitation/hygiene personnel who collaborate in providing critical services to affected populations during health emergencies. By the end of 2024, emergency medical teams had been established in nearly every country in the Western Pacific Region, with 16 classified for international deployments, and many national teams developed to strengthen response to domestic emergencies. This analysis is based on published peer-reviewed literature on emergency medical team development and action in health emergencies in the Western Pacific Region, as well as publicly available data on team collaboration and deployment for health emergency response. This analysis considers the global evolution of the World Health Organization Emergency Medical Team Initiative and describes its development in the Western Pacific Region, including how the teams have contributed to emergency response efforts, and the key enabling factors and challenges faced as they develop and respond to emergencies. The analysis concludes by highlighting opportunities for future development, collaboration, research and insights that may be applicable to the global development of emergency medical teams.

## THE EMERGENCY MEDICAL TEAM INITIATIVE

The World Health Organization (WHO) Emergency Medical Team (EMT) Initiative aims to enhance the speed and quality of health emergency response provided by both national and international EMTs, which include deployable field clinics, hospitals and specialist medical teams in disasters, disease outbreaks and other emergencies with significant health consequences. The initiative was established following the January 2010 Haiti earthquake, which saw hundreds of nongovernmental organizations (NGOs), foreign medical teams (FMTs) and individual foreign clinicians deployed to the country. (*1*-*6*) While not the first large-scale disaster with significant international medical engagement, the Haiti earthquake response highlighted significant gaps in coordination with national authorities and in the quality of medical response provided. For instance, some individual clinicians and medical teams were deployed without basic equipment or medications to operate self-sufficiently, and some provided inadequate or inappropriate care. (*4*-*6*) The Haiti response also highlighted significant gaps in quantitative and qualitative data on the responders and the clinical services provided, a lack of standards and accountability to patients and national authorities, and a lack of professionalism in some clinical response actions. (*4*-*6*)

The Haiti earthquake response demonstrated the beneficial impact that FMTs can have during major sudden-onset disasters, with teams caring for thousands of patients and enabling a significant expansion of clinical services alongside a surge in complex presentations. (*4*-*6*) However, it also highlighted the shortcomings of medical response actions in terms of quality of care, scope of practice, self-sufficiency and coordination with national authorities. (*1*-*6*) With the aim of addressing these deficiencies, WHO and the Pan American Health Organization (PAHO) convened a meeting of disaster medicine experts, governmental health emergency focal points and NGO representatives to establish common principles and standards for medical teams deploying to emergencies with significant health consequences. (*3*) This meeting, held in Havana, Cuba, from 7 to 9 December 2010, led to the development and publication in 2013 of the foundational document, *Classification and minimum standards for foreign medical teams in sudden onset disasters*, which became informally known as the FMT Blue Book. (*1*) This WHO publication formalized core principles and minimum standards for FMTs and led to the establishment of a global governance structure, with WHO hosting the FMT secretariat. It also established specific typologies for FMTs based on their scale, scope of practice and complexity of clinical services to be provided. The creation of this typology framework aimed to establish common terminology, understanding and predictability around FMT engagements, which was previously lacking (**Table 1**). It set out minimum staffing based on typology, expected deployment periods (generally at least 2–4 weeks), self-sufficiency requirements and daily reporting requirements. (*1*)

**Table 1:** Emergency medical team typologies, based on the 2021 Blue Book (*2*)

Type	Capability
**Type 1 mobile**	**Provides daylight-hours care for acute trauma and non-trauma presentations, referrals and community-based care. Must be operational within 24 hours of arrival and be able to manage at least 50 outpatient and emergency cases per day for at least 14 days, working in multiple locations (including hard-to-reach populations) for at least 2 weeks. May perform minor procedures in an outpatient setting (e.g. wound debridement); no major surgical procedures.**
**Type 1 fixed**	**Provides daylight-hours care for acute trauma and non-trauma presentations, referrals and community-based care. Must be operational within 24 hours of arrival and be able to manage at least 100 outpatient and emergency cases per day in a fixed location for at least 14 days. May perform minor procedures in an outpatient setting (e.g. wound debridement); no major surgical procedures.**
**Type 2**	**Provides Type 1 services, plus general and obstetric surgery and inpatient care, operating 24 hours/day, ** **7 days/week. Must be operational within 24–36 hours of arrival and be able to manage at least 100 outpatients, one operating theatre and at least 20 inpatients, including at least 7 major or 15 minor surgical operations, per day for at least 3 weeks.**
**Type 3**	**Provides Type 2 services, plus complex surgical and intensive care capacity, operating 24 hours/day, ** **7 days/week. Must be operational within 36–48 hours of arrival and be able to manage at least 100 outpatients, two operating theatres/tables and at least 40 inpatients per day, including at least 15 major or 30 minor surgical operations per day. Must have at least four intensive care beds. Must be able to operate for at least 4 weeks.**
**Specialized care teams**	**Teams that can be embedded into local health-care facilities or with other emergency medical teams, or that can be self-sustained, and that can provide specialized care (e.g. rehabilitation, surgical, highly infectious diseases, etc.).**

*Source:* World Health Organization. (*7*)

**Fig. 1:**
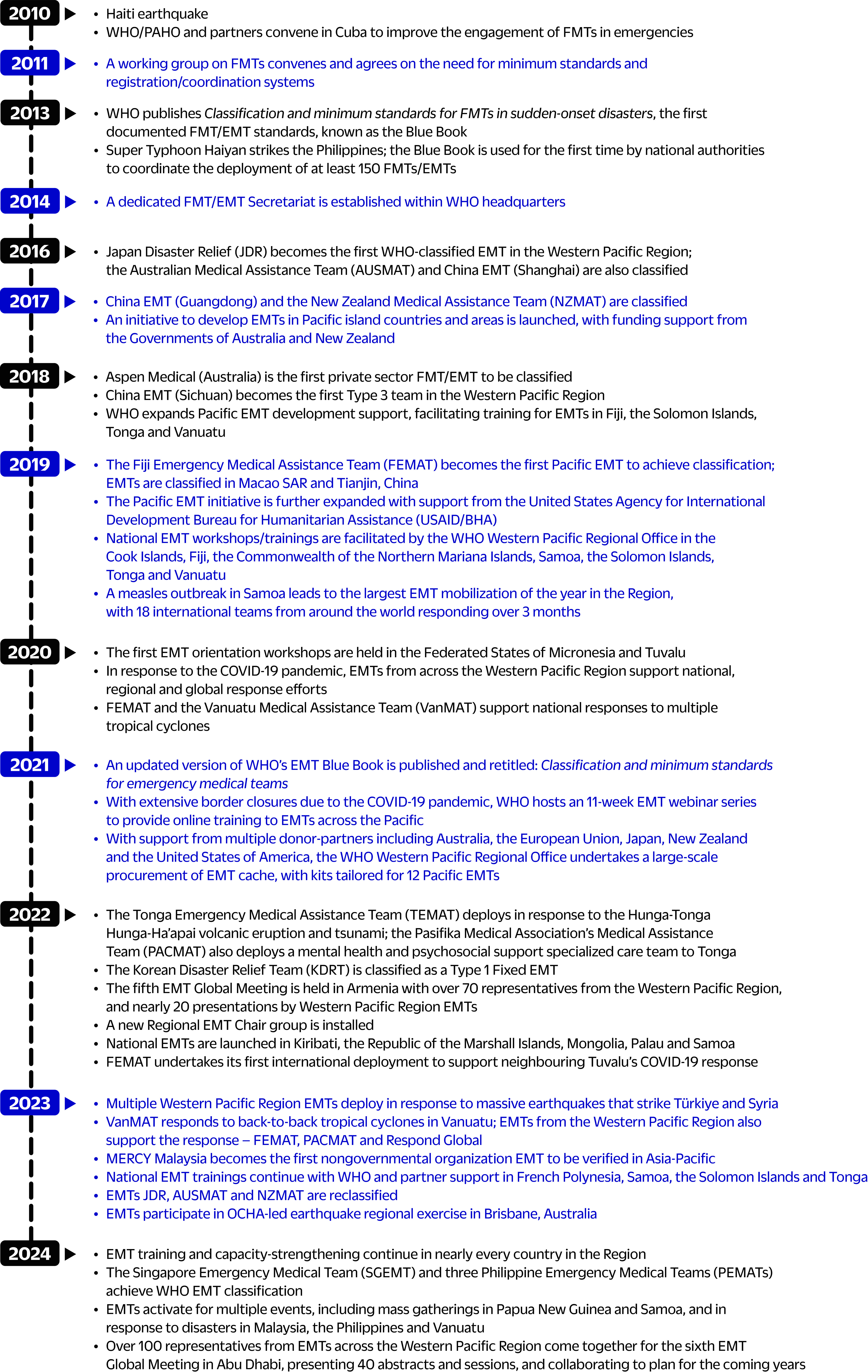
Timeline of key Western Pacific Region EMT events, January 2010–December 2024

In the years following the Haiti earthquake and subsequent meetings to establish FMT standards, teams deployed in response to numerous disasters and outbreaks around the world, including Typhoon Haiyan in the Philippines in 2013 – where the Blue Book was applied for the first time in international EMT coordination – the West Africa Ebola outbreak in 2014–2015, Tropical Cyclone Winston in Fiji in 2016, and many others (**Fig. 1**). (*8*-*18*) During this period, alongside the development of additional formal medical teams by governments and NGOs, WHO established more robust global and regional secretariat functions, such as facilitating FMT development according to established standards and quality assurance of these teams through a process called EMT classification, and supporting nationally led coordination in emergencies. WHO did not set out to establish its own FMTs but worked to coordinate a global network of predictable and coordinated teams capable of providing high-quality care, even in challenging outbreaks or disaster conditions. More national medical teams also emerged in the years that followed the development of formal FMT standards, adopting and adapting the WHO Blue Book principles and standards, including predictable typology (**Table 1**). (*19*, *20*)

[**insert** Figure 1]

In 2015, the language of “foreign medical teams” evolved into “emergency medical teams,” recognizing that national actors are always the first to respond and that strengthening national clinical health emergency response capacity was essential. (*21*) This priority was reflected at the highest level through WHO Executive Board 146, Resolution 10 (2020), which called for “the development of effective and high-performing national, subnational, and regional Emergency Medical Teams, as appropriate, in line with WHO classification and minimum standards.” (*22*) In 2021, WHO’s EMT secretariat, building on nearly a decade of FMT/EMT development, through extensive global consultation, and with the consent of the EMT Strategic Advisory Group (EMT SAG) comprising EMT representatives from the six WHO regions, published updated principles and standards in a new EMT Blue Book entitled, *Classification and minimum standards for emergency medical teams*. (*2*)

The EMT secretariat is based in WHO headquarters in Geneva, Switzerland. It oversees the global classification process of EMTs with each of WHO’s six regional offices in Africa, the Americas, the Eastern Mediterranean, Europe, South-East Asia and the Western Pacific. (*23*) The secretariat focuses on EMT governance, standard-setting, coordination of EMT classification and emergency response coordination. Within WHO regional offices, EMT regional focal points engage in EMT development and training, participate in EMT classification, facilitate information management, and enable regional EMT networks and collaboration (**Tables 2**–**4**). (*23*) Regional focal points also engage with ministries of health and WHO country offices, ensuring coordination across the three levels of the Organization. Financial and technical support from national, regional and international donor-partners enable EMT secretariat functions. These donor-partners sometimes fund and/or provide staffing to national EMT coordination structures in emergencies, support staffing for the WHO EMT secretariat, enable regional and global meetings, support EMT mentoring and classification, and enable work to develop national and subnational EMTs, particularly in low- and middle-income countries (LMICs) and areas. (*16*, *19*, *20*)

**Table 2:** WHO-classified and reclassified^a^ EMTs in the Western Pacific Region in order of verification date, as of 31 December 2024 (*7*)

EMT	Base (city, country)	Type	Year classified (reclassified)
**Japan Disaster Relief (JDR)**	**Tokyo, Japan**	**T1F/M, T2, SCT**	**2016 (2023)**
**China International EMT (Shanghai)**	**Shanghai, China**	**T2**	**2016**
**Australia Medical Assistance Team (AUSMAT)**	**Darwin, Australia**	**T2, T1F, T1M and SCT (surgical and outbreak)**	**2016 (2023)**
**China International EMT (Guangdong)**	**Guangzhou, China**	**T2**	**2017**
**New Zealand Medical Assistance Team (NZMAT)**	**Auckland, New Zealand**	**T1F**	**2017 (2023)**
**Aspen Medical**	**Australia**	**SCT**	**2018**
**China International EMT (Sichuan)**	**Chengdu, China**	**T3**	**2018**
**China International EMT (Macau)**	**Macao SAR, China**	**T1F**	**2019**
**Fiji Emergency Medical Assistance Team (FEMAT)**	**Suva, Fiji**	**T1F**	**2019**
**China International EMT (Tianjin)**	**Tianjin, China**	**T2**	**2019**
**Korea Disaster Relief Team (KDRT)**	**Seoul, Republic of Korea**	**T1F**	**2022**
**MERCY Malaysia**	**Kuala Lumpur, Malaysia**	**T1F**	**2023**
**Singapore Emergency Medical Team (SGEMT)**	**Singapore**	**T1F**	**2024**
**Philippines Emergency Medical Assistance Team (PEMAT), Visayas**	**Tacloban, Philippines**	**T1F**	**2024**
**PEMAT, Metro Manila**	**Metro Manila, Philippines**	**T1F**	**2024**
**PEMAT, Luzon**	**Pampanga, Philippines**	**T1F**	**2024**

EMT: emergency medical team; SCT: specialized care team; SAR: Special Administration Region; T1F: Type 1 fixed; T1M: Type 1 mobile; T2: Type 2; T3: Type 3.

^a^ Classified EMTs should undergo reclassification – or additional peer review/quality assurance 5 years after initial classification.

*Source:* World Health Organization. (*7*)

**Table 4:** EMTs in the Western Pacific Region pursuing WHO classification, as of 31 December 2024

EMT (host country/organization)	Base (city, country)	Type
**Cambodia EMT**	**Phnom Penh, Cambodia**	**T1F**
**China International EMT (Hunan)**	**Changsha, China**	**T2**
**China International EMT (Zhejiang)**	**Hangzhou, China**	**T2**
**Fiji Emergency Medical Assistance Team (FEMAT)**	**Suva, Fiji**	**T1M**
**Humanitarian Medical Assistance (HuMA)**	**Tokyo, Japan**	**T1M**
**Mongolia EMT**	**Ulaanbaatar, Mongolia**	**T1F**
**Pasifika Medical Assistance Team (PACMAT/Pasifika Medical Association)**	**Auckland, New Zealand**	**T1M**
**Peace Winds (Japan)**	**Tokyo, Japan**	**T1M**
**Respond Global**	**Noosa, Australia**	**T1M/SCT**
**Tokushukai Medical Assistance Team (TMAT)**	**Tokyo, Japan**	**T1M**

EMT: emergency medical team; SCT: specialized care team; T1F: Type 1 fixed; T1M: Type 1 mobile; T2: Type 2.

**Table 3:** EMTs in the Western Pacific Region receiving direct support from the WHO Western Pacific Regional Office (national and international)

EMT	City, country	Type	Year established	Status/notes
**Cambodia EMT (CAM-EMT)**	**Phnom Penh, Cambodia**	**T1F**	**2023**	**SOPs drafted. Cache on hand. Pursuing classification as of October 2024.**
**Cook Islands Medical Assistance Team (KukiMAT)**	**Rarotonga, Cook Islands**	**T1M (Pacific adaptation)**	**2019**	**SOPs established. Roster established with several team member trainings and exercises completed. Cache on hand.**
**French Polynesia EMT**	**Tahiti, French Polynesia**	**T1M**	**2023/2024**	**SOPs in development. EMT orientation workshop held in December 2023.**
**Kiribati Medical Assistance Team (KiriMAT)**	**South Tarawa, Kiribati**	**T1M (Pacific adaptation)**	**2022**	**SOPs established and under revision. Roster established with one team member training held (2022).**
**Lao People's Democratic Republic EMT**	**Vientiane, Lao People's Democratic Republic**	**T1F**	**2023**	**SOPs developed. Roster established with several team member trainings (2023, 2024). Cache on hand.**
**Marshall Islands Medical Assistance Team (MI-MAT)**	**Majuro, Republic of the Marshall Islands**	**T1M (Pacific adaptation)**	**2022**	**SOPs established. Roster established with two team member trainings held (2022, 2024).**
**Federated States of Micronesia EMT (FSM EMT)**	**Federated States of Micronesia**	**T1M (3)**	**2019**	**SOPs drafted. Rosters established in two States, with multiple team member trainings held (2023, 2024).**
**Mongolia Global Emergency Response EMT (M-GER EMT)**	**Ulaanbaatar, Mongolia**	**T1F**	**2022**	**SOPs in development. Specialized (winterized) cache on hand.**
**KLEMAT (Palau EMT)**	**Koror, Palau**	**T1M (Pacific adaptation)**	**2022**	**SOPs developed. Roster established with several team member trainings (2022, 2023, 2024). Cache on hand.**
**Papua New Guinea EMT (PNG EMT)**	**Port Moresby, Papua New Guinea**	**T1M**	**2023**	**SOPs developed. Roster established with several team member trainings (2023, 2024). Cache on hand.**
**Samoa Emergency Medical Assistance Team (SEMAT)**	**Apia, Samoa**	**T1M (Pacific adaptation**	**2022**	**SOPs developed. Roster established with several team member trainings (2022, 2023, 2024). Cache on hand.**
**Solomon Islands Medical Assistance Team (SOLMAT)**	**Honiara, Solomon Islands**	**T1M (Pacific adaptation)**	**2018**	**SOPs developed. Roster established with several team member trainings. Cache on hand. Multiple national deployments completed.**
**Tonga Emergency Medical Assistance Team (TEMAT)**	**Nuku’alofa, Tonga**	**T1M (Pacific adaptation)**	**2018**	**SOPs developed. Roster established with several team member trainings (2018, 2019, 2023, 2024). Cache on hand. One national deployment completed.**
**Tuvalu EMT**	**Tuvalu**	**T1M (Pacific adaptation)**	**–**	**National EMT orientation workshop held in January 2020. Further progress limited.**
**Vanuatu Medical Assistance Team (VanMAT)**	**Port Vila, Vanuatu**	**T1M (Pacific adaptation)**	**2018**	**SOPs developed. Roster established. Cache on hand. Multiple team member trainings held, and multiple national deployments completed.**
**Viet Nam EMT**	**Hanoi, Viet Nam**	**T1F**	**2024**	**National EMT orientation workshop held in 2024.**

EMT: emergency medical team; SOP: standard operating procedure; T1F: Type 1 fixed; T1M: Type 1 mobile; WHO: World Health Organization.

The process to quality-assure EMTs for international deployments, known as EMT classification, began in earnest in 2015 with the first EMTs undergoing structured mentoring and external peer review, also known as EMT verification. (*1*, *2*, *7*) This peer-review process, based on the principles and standards detailed in the EMT Blue Book, was established to ensure that EMTs provide high-quality clinical services to patients, are able to function with a high degree of self-sufficiency, and provide receiving countries and ministries of health with a high level of predictability when EMTs deploy. (*1*, *2*) In 2016, the first year in which EMTs achieved classification, seven international teams were verified/classified, including Type 1, 2 and 3 teams in WHO’s European and Western Pacific regions (**Table 2**). (*7*) The classification process included mentorship by peer EMTs, as well as multiple check-in points before verification, to ensure that teams were prepared for their final verification process. As of 2024, no EMT had failed verification. The same standards apply to all teams, regardless of the financial or human resources a government or NGO has available. (*1*, *2*) Several teams have been in the mentorship phase for several years, for instance, due to operational and logistical challenges, financial and human resources constraints, and the impacts of the COVID-19 pandemic. To date, there are no published data regarding the number of EMTs that have initiated but not completed the EMT classification process; this represents an opportunity for future analysis and research.

While continuing to mentor and undertake quality assurance of EMTs for international classification and deployments, the initiative also supports the development of national and subnational EMTs and strengthens the capacity of national health systems to lead the coordination and activation of the response in disasters, outbreaks and other emergencies. (*19*, *20*) The EMT Initiative aims to support governments, NGOs and other health emergency responders to strengthen health emergency surge capacities and workforces, improving response to emergencies and strengthening global health security. (*1*, *2*, *7*, *24*-*28*)

EMTs have become a critical part of national, regional and global health emergency workforces, enabling rapid, high-quality and self-sufficient response to health emergencies. EMTs comprise clinical, mental health and public health personnel, including physicians, nurses and allied health specialists, as well as experts in health logistics and emergency water, sanitation and hygiene (WASH). (*1*, *2*) In recent years, more specialized care teams (SCTs) have developed, further expanding the scope and specialization of EMTs responding to health emergencies. (*2*, *7*)

In 2023, the EMT SAG adopted the *Emergency Medical Teams 2030 strategy*, which recognizes the value of international surge capacity while stressing the importance of: developing and maintaining national EMTs; ensuring accessible and quality health services in emergencies, partnerships and operational governance for the EMT network at global and regional levels; standardization and quality assurance at all levels; and strengthening information systems and evidence to continue to improve EMT development and action. (*24*) The strategy aligns with the International Health Regulations (IHR, 2005), World Health Assembly (WHA) Resolution 75.20 Strengthening the Global Architecture for Health Emergency Preparedness, Response and Resilience, WHO’s Triple Billion targets, the United Nations Sustainable Development Goals, the Global Health Emergency Corps Framework, the Asia Pacific Health Security Action Framework, and the “Grand Bargain,” all of which emphasize strengthening health systems and health emergency preparedness and response capacities. (*24*-*31*)

## EMT DEVELOPMENT IN THE WESTERN PACIFIC

The WHO Western Pacific Region comprises 37 countries and areas (until the addition of Indonesia in May 2025), with some of the world’s largest and smallest countries by population, and with diverse economies and geographies, many of which face a wide range of hazards, including earthquakes, volcanoes, tsunamis, cyclones/typhoons, and a broad array of infectious hazards. (*32*) Every year, millions of people in the Region face a wide array of health emergencies. Some countries in the Western Pacific Region have world-class national resources and capacities, some are emerging and rapidly developing economies, while others face significant and persistent financial, material and human resource constraints. (*16*, *19*, *20*) Recognizing the unique risk profile of the Region and the importance of timely and quality response to health emergencies, many countries and areas across the Region have committed to applying the EMT methodology to save lives and relieve suffering in emergencies (**Tables 2**–**4**). At the same time, several countries/areas have adopted and adapted global standards to their specific contexts, recognizing unique country risk profiles, human resource pools and financial resources availability. (*19*, *20*)

EMTs in the Western Pacific Region frequently engage in capacity development and health emergency response, contribute to the development of EMT standards, and engage in global and regional EMT governance. The Executive Director of Australia’s National Critical Care and Trauma Response Centre, which houses the Australian Medical Assistance Team (AUSMAT), served as the global EMT SAG Chair from 2021 to 2024, and AUSMAT was among the first EMTs to be classified for international response. (*7*, *33*) EMTs from the Region contribute clinical and logistics mentors to support the development of other teams pursuing classification, and they support the development of emerging national teams in the Region and beyond. (*16*, *19*, *20*) In addition, Japan’s EMT, Japan Disaster Relief (JDR), through its secretariat in the Japan International Cooperation Agency (JICA), has worked as a key partner to collect, consolidate and analyse clinical data from EMT response actions, and led the development of the EMT Minimum Data Set (MDS). (*34*-*39*) EMTs in the Region also contribute to EMT coordination training and are consistently represented in EMT technical working groups to expand and refine clinical and operational standards to strengthen EMT action in emergencies. (*40*-*42*)

In the Western Pacific Region, EMTs have expanded in number, scope, capability and interoperability since the genesis of the EMT Initiative (**Tables 2**–**4**). (*7*, *19*, *20*) The Region was home to 16 of the 52 classified international EMTs as of 31 December 2024, including: SCTs capable of providing specialized surge support in surgery, haemodialysis and outbreak response; mobile Type 1 teams that can reach remote islands and villages in a disaster or outbreak; fixed Type 1 teams that can replace or supplement damaged or destroyed health centres and provide emergency and outpatient care; and larger Type 2 and Type 3 EMTs capable of providing inpatient, surgical and specialized care in large-scale emergencies, including intensive/critical care. (*1*, *2*, *7*) Ten EMTs in the Region are pursuing EMT classification (as of 31 December 2024) (**Table 4**). EMTs have been established in the largest and smallest countries, by governments and NGOs, and have responded to emergencies in the Region and around the world. (*7*, *16*, *17*, *19*, *20*, *43*-*63*)

International EMTs often capture the spotlight in health emergency response, and they play a critical role, as demonstrated by EMTs from the Region responding to emergencies as far away as Liberia, Nepal, Sudan and Türkiye. (*10*, *11*, *13*-*15*) At the same time, national EMTs across the Region have continuously demonstrated that investing in strong local health emergency response capacities has enabled local response when emergencies strike. (*16*, *19*, *20*, *43*-*50*) Unfortunately, national EMT response actions are rarely reported in the literature, limiting the ability to assess the impacts of their engagements.

By the end of 2024, nearly every country in the Western Pacific Region had established at least one EMT or engaged with the WHO EMT Initiative to establish national and/or international EMT capability. (*7*, *16*, *19*, *20*) Larger, higher-income countries such as Australia, China and Japan have established sophisticated international EMTs with some of the most advanced capabilities, including complex surgical and intensive-care capacity. (*7*, *64*-*69*) Several these countries have also established networks of thousands of local clinical response teams, sometimes known as Disaster Medical Assistance Teams, designed specifically for response to domestic emergencies. (*69*-*71*)

At the same time, LMICs, including some of the smallest Pacific island countries and areas (PICs), have also taken steps to establish international or national EMTs in recent years, often with financial and technical support from the WHO Western Pacific Regional Office and regional donor-partners. (*16*, *19*, *20*, *72*-*79*) As of 1 November 2024, 14 PICs had already established EMTs or were in the process of doing so, training team members, sourcing equipment and supplies (known as “EMT cache” – many through bulk procurement by the Western Pacific Regional Office leveraging donor-partner financing), and developing standard operating procedures (SOPs) for emergency activations. (*19*, *20*, *76*-*78*) Several countries within the Association of South-East Asian Nations (ASEAN) also took action to develop EMTs, with WHO-classified EMTs in Malaysia, the Philippines (3) and Singapore by the end of 2024. (*58*-*61*, *80*-*85*) Several more are being developed, with at least one seeking international classification.

### Responding at home and around the world: Western Pacific Region EMTs

EMTs have proven to be central to health emergency response in many countries across the Western Pacific Region. (*16*) Even before the global EMT classification process, EMTs from the Region responded to large-scale disasters, such as Typhoon Haiyan (locally named Yolanda) in the Philippines in 2013, Tropical Cyclone (TC) Pam in 2015 in Vanuatu, and TC Winston in Fiji in 2016, among others (**Table 5**). (*8*, *9*)

**Table 5:** EMT activations/deployments in/from the Western Pacific Region (January 2010–December 2024, non-exhaustive)

Year	EMT	Location	Event
**2010**	**AUSMAT, JDR**	**Pakistan**	**Flooding**
	**KDRT, MERCY Malaysia, TMAT**	**Haiti**	**Earthquake**
	**MERCY Malaysia**	**Malaysia**	**Flooding**
	**MERCY Malaysia, TMAT**	**Chile**	**Earthquake**
	**MERCY Malaysia**	**Indonesia**	**Earthquake and tsunami**
	**MERCY Malaysia**	**Pakistan**	**Flooding**
**2011**	**AUSMAT**	**New Zealand**	**Christchurch earthquake**
	**TMAT**	**Japan**	**Great East Japan Earthquake**
	**MERCY Malaysia**	**Malaysia**	**Flooding**
	**MERCY Malaysia**	**Libyan Arab Jamahirya**	**Conflict**
	**MERCY Malaysia**	**Somalia**	**Conflict**
**2012**	**MERCY Malaysia**	**Malaysia**	**Flooding**
	**MERCY Malaysia**	**Philippines (Cagayan de Oro)**	**Tropical storm**
	**MERCY Malaysia**	**Philippines (Davao)**	**Typhoon Bopa**
**2013**	**AUSMAT, China, JDR, KDRT, NZMAT, TMAT**	**Philippines**	**Typhoon Haiyan**
	**AUSMAT, NZMAT**	**Solomon Islands**	**Dengue fever outbreak**
	**AUSMAT**	**Australia (Manigrida, NT)**	**Trachoma outbreak**
	**MERCY Malaysia**	**Malaysia**	**Flooding, conflict**
	**MERCY Malaysia**	**Philippines (Leyte, Bohol)**	**Typhoon Haiyan, earthquake**
**2014**	**China**	**Liberia**	**West Africa Ebola outbreak**
	**NZMAT**	**Solomon Islands**	**Severe flooding**
**2015**	**AUSMAT, JDR, NZMAT, PACMAT**	**Vanuatu**	**Tropical Cyclone Pam**
	**AUSMAT, JDR, KDRT, MERCY Malaysia, TMAT**	**Nepal**	**Earthquake**
**2016**	**AUSMAT, NZMAT, PACMAT**	**Fiji**	**Tropical Cyclone Winston**
	**MERCY Malaysia**	**Indonesia (Aceh)**	**Earthquake**
	**TMAT**	**Japan**	**Kumamoto earthquake**
	**TMAT**	**Haiti**	**Hurricane Matthew**
**2017**	**MERCY Malaysia**	**Malaysia**	**Flooding**
	**MERCY Malaysia**	**Bangladesh**	**Flooding**
	**MERCY Malaysia**	**Sri Lanka**	**Flooding and landslides**
**2018**	**AUSMAT**	**Indonesia, Thailand, Papua New Guinea, Bangladesh**	**Earthquake, tsunami, cave rescue, diphtheria outbreak**
	**KDRT**	**Lao People's Democratic Republic (the)**	**Flooding**
	**AUSMAT, PACMAT**	**Tonga**	**Tropical Cyclone Gita**
	**MERCY Malaysia**	**Indonesia, Lao People's Democratic Republic (the), Malaysia**	**Flooding, earthquake, dam break/flooding**
**2019**	**AUSMAT**	**New Zealand, Australia**	**White Island eruption, bushfires**
	**JDR**	**Mozambique**	**Tropical Cyclone Idai**
	**18 international EMTs**	**Samoa**	**Measles outbreak**
	**China International EMT**	**China**	**Earthquake, mudslide, water penetration**
	**FEMAT**	**Fiji**	**Measles outbreak, Cyclones Sarai and Tino**
	**SOLMAT**	**Solomon Islands**	**Rennel oil spill**
	**MERCY Malaysia**	**Malaysia (Johor)**	**Chemical spill**
**2020**	**China International EMT (several)**	**China, Italy, Algeria, Sudan**	**COVID-19**
	**PEMAT**	**Philippines**	**Taal volcanic eruption, Typhoon Ambo, COVID-19**
	**FEMAT**	**Fiji**	**COVID-19, Cyclones Harold and Yasa**
	**AUSMAT**	**Japan, China, Australia (various locations)**	**COVID-19**
	**SOLMAT**	**Solomon Islands**	**COVID-19**
	**MERCY Malaysia**	**Malaysia**	**COVID-19**
**2021**	**China International EMT (several)**	**China**	**Various earthquakes, social security incidents**
	**FEMAT**	**Fiji**	**COVID-19, Tropical Cyclone Ana**
	**AUSMAT, NZMAT**	**Fiji, Australia (various locations)**	**COVID-19**
	**PEMAT**	**Philippines**	**Typhoon Odette**
	**MERCY Malaysia**	**Malaysia**	**COVID-19**
	**NZMAT**	**Cook Islands**	**COVID-19**
**2022**	**PACMAT, TEMAT**	**Tonga**	**Hunga-Tonga eruption and tsunami**
	**China International EMT**	**China (Sichuan, Tibet)**	**Earthquakes, avalanche**
	**AUSMAT**	**Australia, multiple Pacific island countries (Kiribati, Solomon Islands, Vanuatu)**	**COVID-19**
	**PEMAT**	**Philippines (Northern Luzon)**	**Earthquake**
	**FEMAT, NZMAT, PACMAT**	**Niue, Fiji**	**COVID-19**
**2023**	**PACMAT**	**New Zealand**	**Tropical Cyclone Gabrielle**
	**JDR, Mongolia Military EMT, PEMAT, TMAT**	**Türkiye**	**Earthquake**
	**FEMAT, PACMAT, Respond Global, VanMAT**	**Vanuatu**	**Tropical Cyclones Judy and Kevin**
	**MERCY Malaysia, MIMAT**	**Pacific Islands, Malaysia**	**COVID-19**
**2024**	**Peace Winds Japan, TMAT**	**Japan**	**Earthquake**
	**PNG EMT**	**Papua New Guinea**	**Papal visit**
	**NZMAT, SEMAT**	**Samoa**	**Commonwealth Heads of Government meeting**
	**PEMAT**	**Philippines**	**Multiple typhoons**
	**MERCY Malaysia**	**Malaysia**	**Flood response**
	**AUSMAT, FEMAT, JICA,a NZMAT, PACMAT, Respond Global, VanMAT**	**Vanuatu**	**17 December 7.3 offshore earthquake**

AUSMAT: Australian Medical Assistance Team; EMT: emergency medical team; FEMAT: Fiji Emergency Medical Assistance Team; JDR: Japan Disaster Relief; JICA: Japan International Cooperation Agency; KDRT: Korea Disaster Relief Team; MIMAT: Marshall Islands Medical Assistance Team; NT: Northern Territory of Australia; NZMAT: New Zealand Medical Assistance Team; PACMAT: Pasifika Medical Assistance Team; PEMAT: Philippine Emergency Medical Assistance Team; PNG EMT: Papua New Guinea Emergency Medical Team; SEMAT: Samoa Emergency Medical Assistance Team; SOLMAT: Solomon Islands Medical Assistance Team; TEMAT: Tonga Emergency Medical Assistance Team; TMAT: Tokushukai Medical Assistance Team; VanMAT: Vanuatu Medical Assistance Team.

^a^ Data/information management support.

In 2019, the largest EMT activation in the Region was mounted in the South Pacific country of Samoa, where 18 international EMTs deployed in response to a measles outbreak that swept across the country. (*17*) This response, led by Samoa’s Ministry of Health, integrated EMTs from around the world into Samoa’s hospitals and health centres and established standalone, spillover clinical areas, significantly expanding intensive, high-dependency and general bed capacity. EMTs also supported Samoa’s large-scale national vaccination campaign, helping to end the outbreak. (*17*) While EMTs typically deploy for 2–4 weeks, several of them extended their deployments to Samoa by several months, requiring multiple team member rotations and resupply of medicines and consumables from their countries of origin. EMT composition, based on EMT Blue Book typology and standards, was partially designed for disaster response, and required adaptation to Samoa’s outbreak scenario (**Table 1**). (*17*)

Throughout the COVID-19 pandemic response, international EMTs deployed to provide technical and operational response support. (*45*, *50*, *64*, *68*) Teams from Australia and New Zealand played particularly important roles in supporting national response efforts in the South Pacific, with multiple deployments reinforcing local capacities in case management, infection prevention and control, vaccination and health logistics in Cook Islands, Fiji, Niue and the Solomon Islands. (*50*, *86*) China International EMT (Macao) deployed to Algeria and Sudan to support early COVID-19 response efforts. (*87*)

In 2022, EMT activations in the Region highlighted the importance of this diversified portfolio of national and international teams of various sizes and capabilities. In January 2022, the Tonga Emergency Medical Assistance Team (TEMAT) deployed independently to the island kingdom’s Ha’apai island group following a volcanic eruption and tsunami that damaged infrastructure and led to several deaths. (*43*, *44*) In 2023, the Vanuatu Medical Assistance Team (VanMAT) deployed in response to back-to-back tropical cyclones. (*62*) Several EMTs from the Western Pacific Region deployed to Türkiye in response to multiple earthquakes, including EMTs from Japan, Mongolia and the Philippines. (*15*) In 2024, EMTs deployed to multiple disasters in the Region, including the Philippine Emergency Medical Assistance Teams (PEMATs) in response to six typhoons in 1 month in the Philippines, MERCY Malaysia in response to flooding in Malaysia, and multiple EMTs in response to the 7.3 magnitude offshore earthquake that struck the South Pacific nation of Vanuatu in mid-December 2024 (**Table 5**).

International surge capacity remains essential when national capacities are overwhelmed, but strong national EMTs have proven important to rapid response that is adapted to a country’s unique needs and operational contexts (**Table 4**). (*16*, *19*, *20*, *43*-*49*) In recent years, national EMTs have responded more frequently and more independently to emergencies within their countries; unfortunately, these actions are not frequently documented through robust data collection and reporting, or in the academic literature. (*18*, *19*) At the same time, international EMTs in the Western Pacific Region have expanded their technical and operational collaboration with these teams, focusing on capacity development and exchange. (*16*, *19*) Collaborative efforts between national and international EMTs are essential to national, regional and global health security. Together, these capacities strengthen national health emergency response capacities, quickly identify when national capacities are overwhelmed, and enable rapid deployment of quality-assured and interoperable EMTs. Together, these comprise key components of what WHO is terming the Global Health Emergency Corps. (*26*)

The application of EMT principles, standards and coordination mechanisms is now well established in many countries and areas, particularly in the Western Pacific Region. At the subregional level, ASEAN has adopted and applied the EMT coordination methodology through the *Standard operating procedure for regional standby arrangements and coordination of joint disaster relief and emergency response operations* (known as the SASOP). (*83*) National and international EMTs in the Region now ensure a high level of predictability and reliability in health emergency response, including in clinical quality, self-sufficiency, coordination mechanisms and reporting. However, some of these teams continue to rely heavily on donor-partner support and are not yet fully integrated into national emergency plans, systems and structures. (*16*, *19*, *20*) Ensuring the sustainability of national EMT capacities remains challenging, as does objectively documenting their work and impacts. (*24*)

### Enabling EMT development and response action

The development of EMTs in the Western Pacific Region, particularly the development of national teams in LMICs, has been enabled through years of commitment and investment by countries and organizations developing their own EMTs, as well as several partners, including: the Government of Australia through its Department of Foreign Affairs and Trade; the European Union; the Health Bureau of the Government of Macao Special Administrative Region, China; the Government of Japan; the Government of New Zealand through its Ministry of Foreign Affairs and Trade; the Government of the United States through the United States Agency for International Development Bureau for Humanitarian Assistance (through to 20 January 2025); and the World Health Organization. (*16*, *19*, *20*) This support, based on political will and diplomatic commitments, technical exchange and capacity-sharing, in-kind material support and funding, has enabled the network of EMTs across the Region to expand and develop. Some support has also been received through bilateral engagements between individual countries.

Additionally, donor-partners have funded WHO’s Western Pacific Regional Office to support EMT development across the Region. Since 2016, through WHO and bilateral support, donor-partners have invested over US$ 5 million in the development of EMTs across the Region and have played a key enabling role in the expansion of the EMT network. (*19*, *20*) Their support has provided funding for EMT member training, facilitated procurement of appropriate cache for mobile EMTs, including centralized cache procurement for many teams by the Western Pacific Regional Office, and enabled shared learning and continuous improvement across teams. (*16*, *19*, *20*, *72*-*79*) This investment supports the localization of health emergency capacities in line with the Grand Bargain localization commitments made at the 2016 World Humanitarian Summit. (*31*)

### Challenges

While EMT development progress is evident across the Region (**Tables 2**–**4**), and EMT engagement in health emergency response efforts is now occurring consistently, there remain challenges worth noting. National and international EMTs have developed relatively quickly in recent years (**Tables 2**–**4**) across the Region; however, some of these efforts have been heavily reliant on external support. National EMTs are yet to be fully institutionalized in laws, policies and budgets in several countries, and continued reliance on external financial support may compromise the sustainability of EMTs if such support were reduced or withdrawn. (*19*, *20*)

Beyond financial concerns, many national EMTs – especially in smaller PICs – face logistical and operational hurdles. Some lack dedicated personnel to support team deployment readiness, dedicated warehousing to store EMT cache, and codified mechanisms to release funds for rapid deployment, sometimes leading to delayed deployments and risking damage or loss of critical supplies. EMT logistics remains a significantly underresearched area, and this reflects an opportunity for future consideration by EMTs and academics researching EMT actions. (*76*-*79*)

Across the Region, EMTs have been active in health emergency response efforts for more than 14 years (**Table 5**). However, quantifiable data demonstrating the impacts of these deployments in terms of reduced population morbidity and mortality, improved access to quality health services delivery, and the speed of health system recovery remain limited. Efforts are underway to strengthen EMT information and data management, analysis and reporting/visualization, particularly with the support of partners such as JICA/JDR. However, this is an area ripe for additional efforts, investments and operational research to demonstrate quantifiable impacts and utility of EMTs. (*24*, *34*-*39*, *59*)

While the EMT Blue Book, the EMT 2030 strategy and other key frameworks have highlighted the importance of coordinated and predictable EMT response based on medical needs, some variances from these approaches have been noted in recent response efforts. In some cases, international EMTs have anecdotally deployed based on requests at diplomatic rather than technical levels, with receiving ministries of health sometimes learning of EMT deployments after they have been initiated. Some EMTs have also deployed outside of the scope of their EMT classification, potentially compromising predictability, quality and patient safety. Since these matters may be considered diplomatically or politically sensitive, there is little formal reporting or research on such actions. However, efforts should be made to apply EMT standards, including ensuring that teams work within their classified typology and deploy only when requested by appropriate and empowered health officials. This is another area that would benefit from future operational research. (*24*)

## Discussion

Recent health emergencies, including the COVID-19 pandemic, other disease outbreaks, and disasters, have demonstrated the critical roles that national and international EMTs have played within the Western Pacific Region and around the world. At the same time, operational research on EMT deployments remains limited, with significant opportunities to better capture their impact on clinical outcomes and health systems in crisis, in line with the EMT 2030 strategy. (*24*)

Looking forward, EMTs will remain essential to national, regional and global health emergency response, contributing to health security strengthening, limiting morbidity and mortality in major emergencies, easing pain and suffering, and strengthening the containment of outbreaks. The EMT network has grown significantly in recent years, with teams now established in nearly every country of the Region. Opportunities remain for teams to enhance collaboration and interoperability, to continue to learn and improve, to leverage their capabilities for diversified response, including in coordination with other rapid response/surge capacities, and to document their impact through data collection, analysis and publication. (*16*)

The Region has several EMTs already applying the EMT 2030 strategic goal of expanded interoperability. These include PEMAT in the Philippines, which had three EMTs classified within the same week in 2024, with common SOPs and team member training. (*60*) Federated States of Micronesia EMT (FSM EMT) applies a similar principle, with its national and state-level teams working together under a common structure and procedures. The national teams of Federated States of Micronesia are able to respond within their respective states but also to work together in case of a larger national or subregional emergency. (*51*) While many Pacific EMTs have been developed primarily for national response, they have common WHO Western Pacific Regional Office-procured cache, their team members have been trained using a common curriculum with Regional Office support, and their SOPs are based on a common template, facilitating future collaboration. (*19*, *20*, *72*-*78*) Beyond reflecting the EMT 2030 objective, this interoperability, at both national and regional levels, can serve as a useful point of reference for other regions by simplifying EMT development through common goods, such as templated SOPs, facilitating access to EMT cache through bulk procurement for multiple teams, and enabling collaboration, with common training and operational approaches.

While expanding in number, EMTs in the Western Pacific Region also continue to expand in their scope of practice and depth of experience, building on response experience and further refining and expanding capabilities. These teams are largely designed for health emergency response, but they are also actively contributing to global EMT technical working groups, supporting learning and strengthening standards across the global EMT network, and resources for health emergency response more generally. At the EMT Global Meeting in November 2024, EMTs from across the Region reiterated their commitment to supporting one another and to strengthening health emergency response in the Region and around the world.

### Limitations and future research

With progress made in EMT development and coordination, there remains significant scope for ongoing learning, improvement and strengthening the EMT evidence base. The Western Pacific Regional Office has contributed to this effort through the development of this EMT Special Edition of WHO’s *Western Pacific Surveillance and Response* (WPSAR) journal, as well as by supporting the development of abstracts for oral and poster presentations at EMT global meetings and in other fora. However, many EMT development efforts, response actions and lessons from the field go undocumented, underscoring the need for continued efforts and investments in these areas, including collecting and publishing data on clinical presentations, patient outcomes, response times and health system recovery timelines following EMT deployments.

This regional analysis, the first to focus on EMT development and action in the Region, leverages published and publicly available information from EMTs to consolidate data and present the most comprehensive summary to date on the work that has been done, as well as on the opportunities that remain. This article draws on extensive peer-reviewed and other official sources, although a systematic literature review was not undertaken. Further documentation of EMT development and response actions, and the sharing of learnings through these efforts, are reflected in strategic objective 4 of the EMT 2030 strategy and are critical for continued EMT improvements in the future. (*24*)

Teams are already working together frequently, collaborating in training, development and deployments; however, opportunities to strengthen collaborations remain, at the subregional level (for example, ASEAN) and through bilateral collaborations, joint trainings and exercises, and through “twinning” arrangements. Continued investments in national and subnational EMT development can strengthen the speed and quality of national response efforts to emergencies, and potentially also reduce reliance on international EMTs and other responders. Evidence on these kinds of impacts is limited but should continue to be a subject for future research, based on more robust data collection and reporting by EMTs. (*24*)

Several EMTs in the Region have developed or are now developing SCTs, and this effort can continue to be expanded and researched. SCTs, such as for mental health and psychosocial support, may contribute to future emergency response efforts. However, technical standards for several proposed SCTs do not yet exist or are in development, and research on their impacts, both positive and negative, is critical for understanding how to optimize their engagement in future health emergency response efforts.

Finally, very few EMTs from the Region have engaged in response to conflicts, deliberate events, or other events related to chemical, radiological and/or nuclear hazards (**Table 5**). As EMTs continue to evolve, expanding capabilities in these areas will be essential. Future research should focus on developing technical standards, response protocols and training for these emerging challenges.

### Conclusions

EMTs are now a well established and trusted component of health emergency response and form a core component of the global health emergency workforce. This is evidenced by the extensive actions that EMTs have taken in health emergency response efforts, by the investments that governments, NGOs and donor-partners have made in EMT development and quality assurance, and by the active engagement of thousands of individual health workers, logisticians and other EMT members. This regional analysis highlights the achievements of the EMTs and their alignment with the global and regional priorities of Member States, reinforcing their critical role in health emergency preparedness and response.
